# Response to Screening of diabetes mellitus among people living with HIV – a comment on “Diabetes mellitus burden among people living with HIV from the Asia‐Pacific region” (Han et al. 2019)

**DOI:** 10.1002/jia2.25334

**Published:** 2019-06-28

**Authors:** Win M Han, Awachana Jiamsakul, Sasisopin Kiertiburanakul, Jeremy Ross, Anchalee Avihingsanon

**Affiliations:** ^1^ HIV‐NAT/Thai Red Cross AIDS Research Centre Bangkok Thailand; ^2^ The Kirby Institute UNSW Sydney Sydney Australia; ^3^ Faculty of Medicine Ramathibodi Hospital Mahidol University Bangkok Thailand; ^4^ TREAT Asia amfAR ‐ The Foundation for AIDS Research Bangkok Thailand; ^5^ Faculty of Medicine Chulalongkorn University Bangkok Thailand

**Keywords:** diabetes, HIV, screening, viral suppression, Asia‐Pacific

Dear Editor,

We appreciate the opportunity to respond to a *Letter to the Editor* about our recently published article entitled “Diabetes mellitus burden among people living with HIV from the Asia‐Pacific region” 1. The letter notes that the sensitivity of the screening tests used in our study differed substantially, and the author of the letter favoured haemoglobin A1c (HbA1c) and the two‐hour plasma glucose level after an oral glucose tolerance test (OGTT) over fasting blood glucose (FBG). The author also highlighted the discrepancies in the proportions of patients diagnosed with diabetes mellitus (DM) by each of the screening tests used in our study and that most of the DM patients were diagnosed by FBG.

While we fully acknowledge the differing sensitivities of these screening tests in diagnosing DM, we want to emphasize that the data analyzed in our observational cohort study were collected from routine clinical care in participating sites. Not all patients included in our analysis would have undergone all mentioned DM screening tests, as test availability and use have varied across the sites in the cohort over time. FBG has been the most widely accessible test, so it would be expected that FBG data availability was greater than that of HbA1c and OGTT in our cohort. The differences in the number of participants diagnosed by FBG compared to HbA1c and OGTT may reflect the later inclusion of HbA1c and OGTT into our cohort data collection as well as differential costs associated with those tests in the context of routine care. Notably, study investigators did not select which tests would be used *a priori*, and we acknowledge that this as a limitation of the study analysis. However, we believe this does not alter our recommendation for improved monitoring and routine screening of non‐communicable diseases including DM among people living with HIV (PLHIV) on long‐term ART using available laboratory testing.

The letter stated that HbA1c screening identified more cases of DM than FBG in studies in Vietnam 2 and Taiwan 3, we would note that participants from both of these studies were from HIV‐negative populations. More studies are recommending the use of FBG testing as the primary screening tool to diagnose DM among PLHIV due to the potential risk of HbA1c underestimating glycaemia in some PLHIV (e.g. on certain ART regimens, who have a higher mean corpuscular volume of red blood cells and lower CD4 counts) 4, 5, 6, 7. We do agree with the author of the letter that testing beyond FBG would be useful for DM screening among PLHIV who are receiving chronic HIV care, and will monitor test availability and reliability in future studies of cardiometabolic outcomes in our cohort.

## Competing interests

The authors have no conflicts of interest to declare.

## Authors’ contributions

WMH and AJ drafted the response. WMH, AJ, SK, JR and AA reviewed and provided revisions to the draft prior, before WMH circulated the letter for approval from all authors from the original article.
